# Supplementary material for: Neural sentence embedding models for semantic similarity estimation in the biomedical domain
Source: BMC Bioinformatics. 2019 Apr 11;20:178. doi: 10.1186/s12859-019-2789-2 (PMC6460644; doi:10.1186/s12859-019-2789-2)
Supplement: Supplementary file 1 — Supplementary tables and figures. (DOCX 181 kb) [file 12859_2019_2789_MOESM1_ESM.docx]

# Additional file 1

Table S1: Hyper-parameters of PV-DM (r=0.819) and PV-DBOW (r=0.804) models

| **Parameter** | **Explanation** | **Value** |
| --- | --- | --- |
| *size* | Vector dimension | 100 |
| *alpha* | Initial learning rate | 0.025 |
| *window* | Maximum distance between the current and predicted word within a sentence | 5 |
| *min_count* | Minimum count of word occurrences required for a word to be included | 5 |
| *negative* | Negative sampling | 0 |
| *hs* | Hierarchical sampling | 1 |

Table S2: Default sent2vec hyper-parameters^1^, investigated values and values of our best sent2vec model (r=0.798)

| **Hyper-parameter** | **Explanation** | **Default values^1^** | **Investigated values** | **Values of best model** |
| --- | --- | --- | --- | --- |
| *lr* | Learning rate (alpha) | 0.2 | 0.2 | 0.2 |
| *lrUpdateRate* | Update rate of learning rate | 100 | 100 | 100 |
| *dim* | Vector dimension | 100 | 100, 700, 1000 | 1000 |
| *wordNgrams* | Number of word n-grams | 2 | 2, 4 | 2 |
| *epoch* | Number of training epochs | 5 | 5, 9 | 9 |
| *minCount* | Minimum count of word occurrences required for a word to be included in the vectors | 5 | 5, 50 | 50 |
| *dropoutK* | Number of n-grams dropped when training a model | 2 | 2, 4 | 4 |
| *t* | Sampling threshold | 0.0001 | 0.0001, 0,000001 | 0.000001 |
| *neg* | Negative sample size | 10 | 10 | 10 |
| *loss* | Loss of the objective function | ns | ns | ns |

ns…non-saturating

^1^https://github.com/epfml/sent2vec

Table S3: Hyper-parameters of fastText CBOW and skip-gram models

| **Hyper-parameter** | **Explanation** | **Values** | **Investigated values** | **Values of best model (skip-gram / CBOW)** |
| --- | --- | --- | --- | --- |
| *lr* | Learning rate (alpha) | 0.1 | 0.1 | 0.1 / 0.1 |
| *lrUpdateRate* | Update rate of learning rate | 100 | 100 | 100 / 100 |
| *dim* | Vector dimension | 100 | 100, 300 | 100 / 100 |
| *ws* | Size of the context window | 5 | 5, 10 | 5 / 5 |
| *wordNgrams* | Max length of word n-grams | 1 | 1, 2, 3 | 3 / 3 |
| *epoch* | Number of training epochs | 5 | 5 | 5 / 5 |
| *minCount* | Minimum count of word occurrences required for a word to be included in the vectors | 5 | 5, 50 | 50 / 50 |
| *t* | Sampling threshold | 0.0001 | 0.0001 | 0.0001 / 0.0001 |
| *neg* | Negative sample size | 5 | 5 | 5 / 5 |
| *loss* | Loss of the objective function | softmax | softmax | softmax / softmax |

Table S4: Hyper-parameters of skip-thoughts model

| **Hyper-parameter** | **Explanation** | **Values** |
| --- | --- | --- |
| *word_embedding_dim* | Word embedding dimension | 620 |
| *bidirectional_encoder* | Whether to use a bidirectional or unidirectional encoder RNN | False |
| *encoder_dim* | Number of output dimensions of the sentence encoder | 2400 |
| *learning_rate* | Initial learning rate | 0.0008 |
| *learning_rate_decay_factor* | Learning rate decay factor | 0.5 |
| *learning_rate_decay_steps* | The number of steps before the learning rate decays. | 400000 |
| *number_of_steps* | Total number of training steps | 500000 |
| *clip_gradient_norm* | Gradients are clipped to this value | 5.0 |

RNN…Recurrent neural network


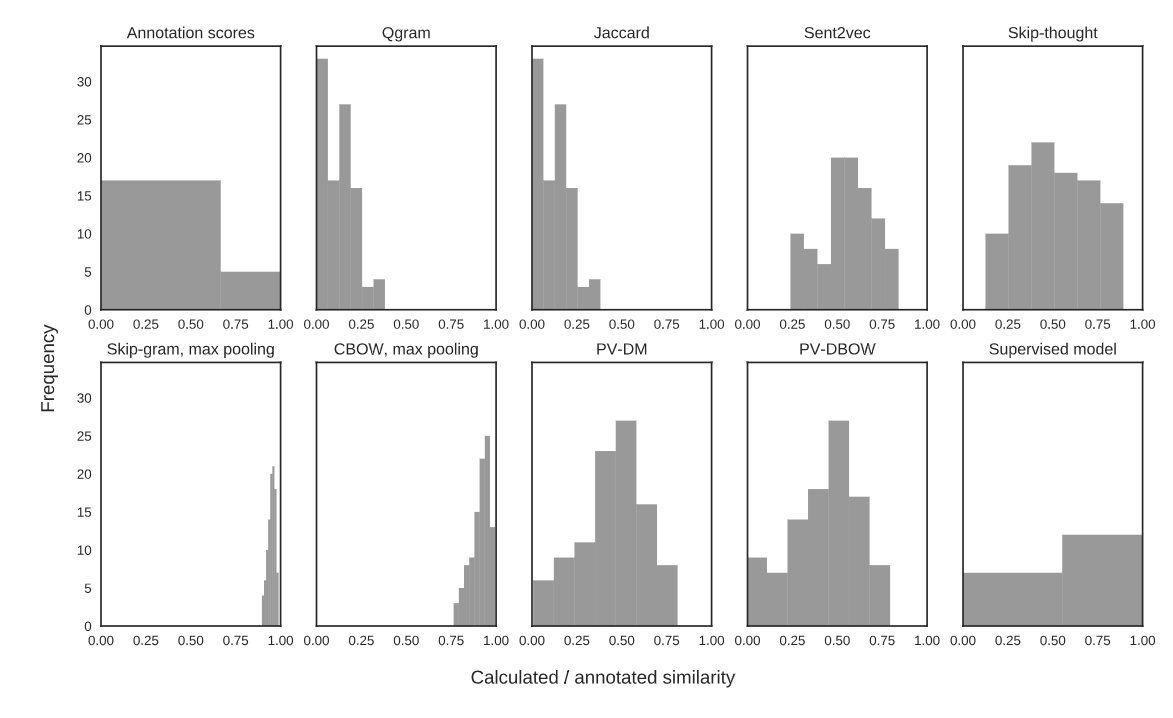


Figure S1: Distribution of annotated and calculated similarity scores of different models


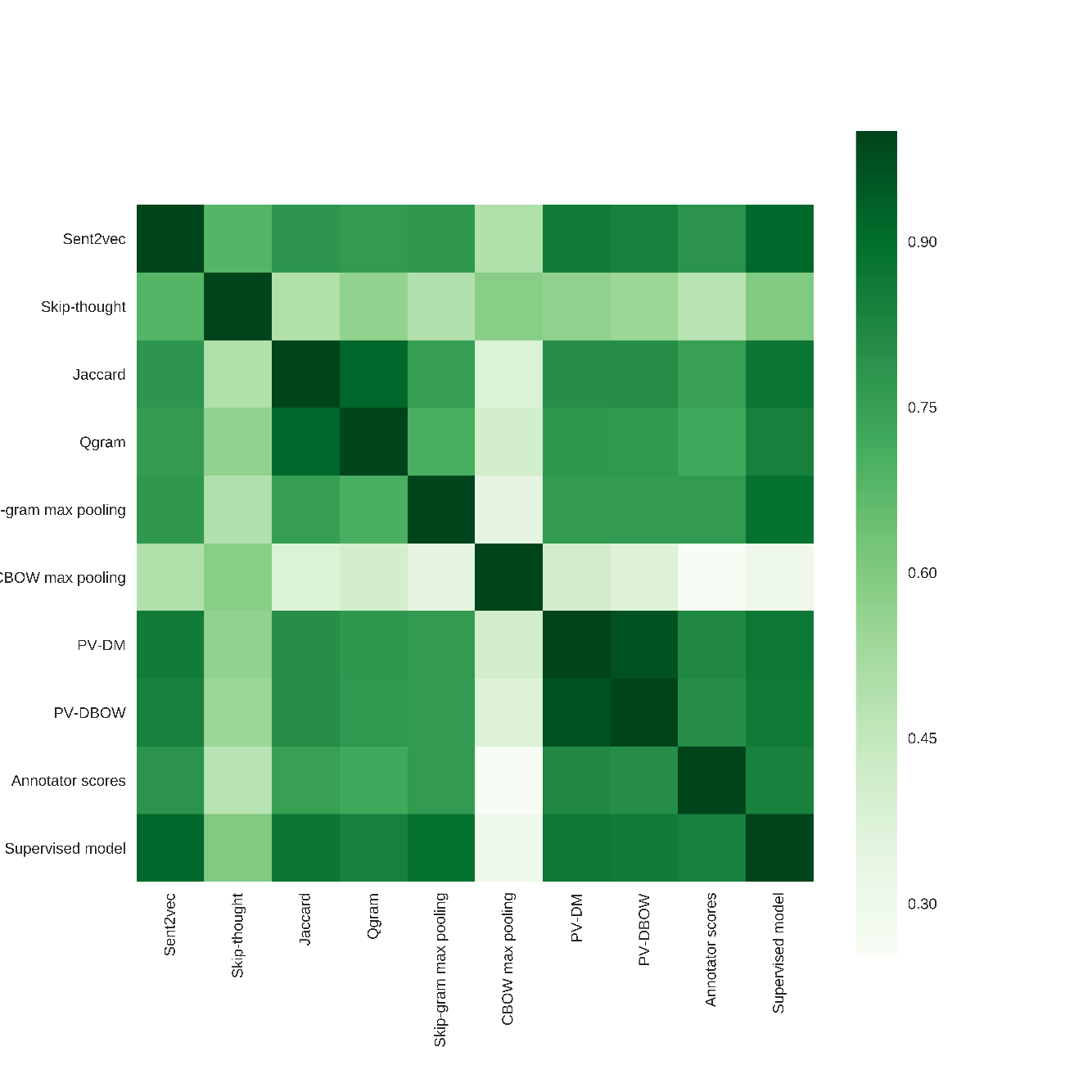


Figure S2: Correlation between different trained models.
